# Microbiome in aging of Gut and Brain (MiaGB): paving the ways to understand gut-brain axis in aging

**DOI:** 10.31491/apt.2022.03.080

**Published:** 2022-03-30

**Authors:** Michal M. Masternak, Hariom Yadav

**Affiliations:** a Burnett School of Biomedical Sciences, College of Medicine, University of Central Florida, Orlando, FL 32827, USA.; b Department of Head and Neck Surgery, Poznan University of Medical Sciences, Poznan, Poland.; c USF Center for Microbiome Research, Microbiomes Institute, Tampa, FL 33612, USA.; d Center for Excellence of Aging and Brain Repair, University of South Florida, Tampa, FL 33612, USA.

**Keywords:** Aging, microbiome, gut, brain, diet, dementia, cognition

## Abstract

Decades of aging research established several well-characterized theories of aging, yet as the studies often focus on different cellular mechanisms there is overall agreement that organismal aging is characterized by multi-factorial degenerative processes resulting from multiple alterations of different molecular pathways compromising cellular or tissues functions. Due to this complexity aging is a major risk factor for multiple diseases including cardiovascular diseases, cancers, diabetes, and neurological diseases such as Alzheimer’s disease. It is well known that this multi-factorial process in some cases might be accelerated by the dysfunction of one organ as a source of chronic low-grade inflammation. Importantly, most recent studies provide strong evidence that the gut microbiome represents a new independent organ system mainly composed of a variety of microorganisms recognized as the microbiome. The high integrity of the microbiome with the host physiology and biochemical interactions between specific bacteria and cellular processes supports its organ-like function in organismal health and the process of aging. However, it is important to better understand what causes potential cellular stress to accelerate a variety of pathological changes, what is the specific role of our gut microbiome in process of human aging, and how we could use this knowledge to prevent or delay aging pathology.

## Gut microbiome and aging

Recently, there is increasing interest in the potential role of gut bacteria in process of aging and especially the connection between the gut microbiome and healthy brain function. Importantly, the human body harbors an astonishing number (around 40 to 100 trillion) of bacteria, which outnumber the number of cells in the human body [1]. This combination of commensal, symbiotic, and pathogenic microorganisms is known as the microbiome, which is known to maintain organismal health by regulating fiber catabolism, biosynthesis of amino acids and vitamins, xenobiotics detoxification, resistance to pathogens, and modulation of the host immune system [2]. However, the gut microbiota composition can fluctuate due to different environmental/dietary and internal/organismal conditions.

Already in early 1900, a biologist Elie Metchnikoff observed that centenarians living in the Bulgarian regions were consuming fermented milk foods, which he associated with increased longevity and healthy aging in this specific population [3]. Later, those bacteria fermenting milk products have emerged as probiotics (pro-life). For a long time, the diet was accepted as the most influential factor modulating the composition of microbial flora in the gut. Thus, consuming probiotic-rich foods can improve gut microbiota by balancing “good” *vs*. “bad” bacteria families, providing extended protection from leaky gut and exposure to pathogenic bacteria, while antibiotic interventions destroy gut microbiota leaving it unprotected and disturbing all metabolic processes in our digestive system, which needs time to re-establish its previous conditions.

Interestingly, despite strong associations between microbiota and consumption of probiotic-rich foods, the diet and environment are not the only factors that influence the microbiota. Recently, there is strong evidence that genetics play important role in the establishment of a healthy gut microbiome. The studies with long-living Ames dwarf mice, the animals that are genetically predisposed to living longer and healthier lives indicated that despite being born from the same parents, living under the identical pathogen-free environment, and consuming identical food, they develop different microbiota when comparing not only fecal samples but also the content of ileum, cecum or distal colon [4]. Additionally, subjecting animals to calorie restriction (CR) one of the most powerful non-pharmacological interventions known to extend the lifespan in different animal models without changing the composition of the diet significantly alters the composition of gut microbiota promoting the increase of “good” bacteria over the “bad” one [4]. Also, reducing inflammation in the gut tissue by eliminating senescent cells using senolytic drug treatment also promotes healthy gut microbiome [5]. More importantly, enhancement of genetic studies by incorporating and enhancing Next Generation Sequencing analysis during the last decade increased the scientific evidence that alterations in the diversity of the gut flora and reduction in the abundance of beneficial bacteria can impair the function of many different organs and have a negative impact of healthspan and lifespan. In the elderly population, microbial dysbiosis results in the accumulation of life-threatening bacteria disturbing intestinal homeostasis and the development of several systemic diseases as well as premature mortality [6].

## Gut microbiota in Alzheimer’s disease (AD) and AD-related dementias (ADRD)-MiaGB Study

**Figure 1.**
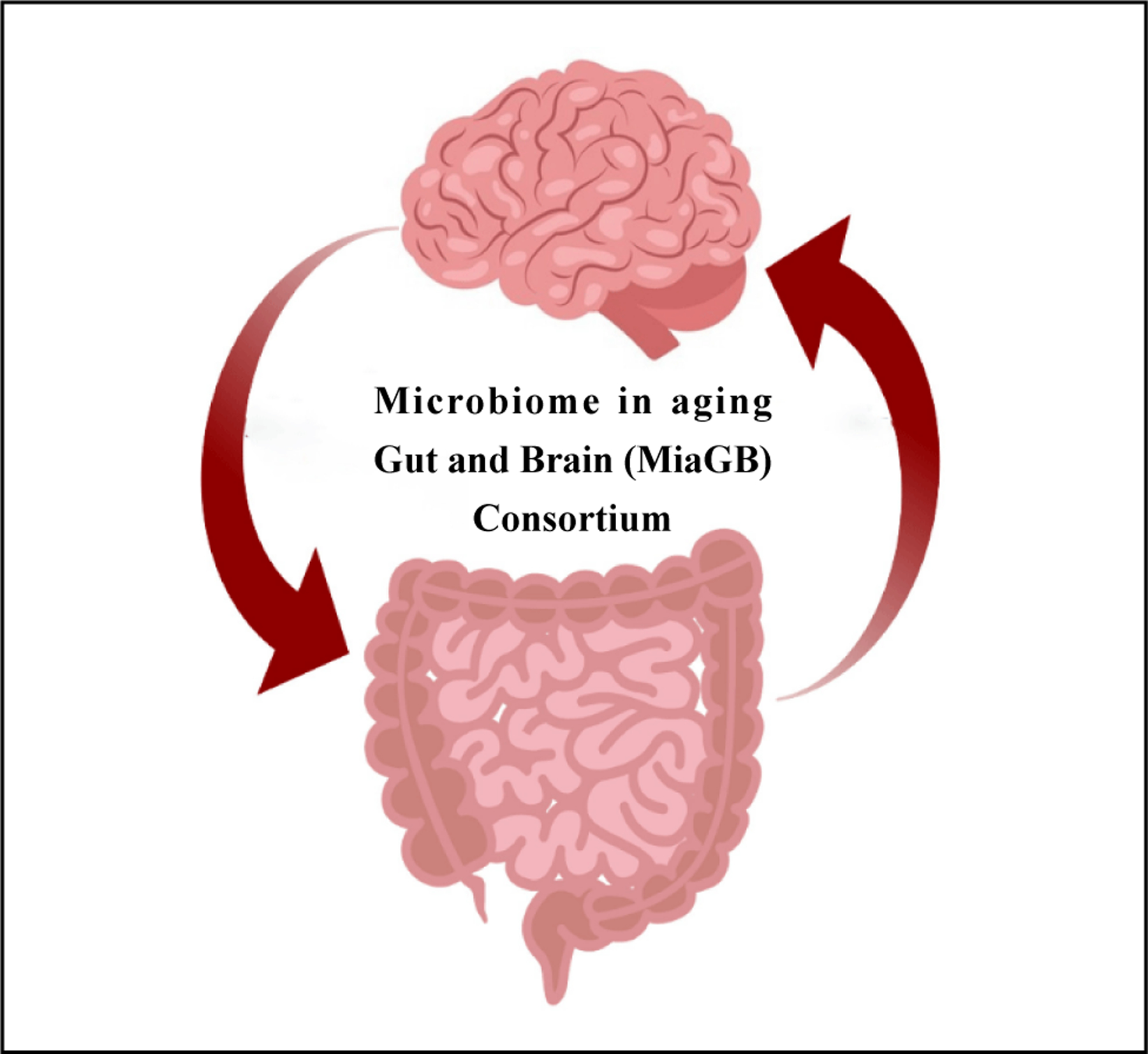
Study of Microbiome in aging Gut and Brain (MiaGB) Consortium deciphering how gut health contributes to brain functions during human aging.

AD is the sixth leading cause of death in the U.S. and the mechanisms responsible for AD remain poorly understood. Studies have identified many genes associated with the risk of development of AD, however, few have examined how the underlying mechanisms involved with longevity and health span might influence AD risk and progression. Importantly, there is also a significant emerging interest in studying the impact of gut microbiota on cognitive function and the development of AD. Interestingly, we and others found that the gut microbiome signature in older adults with mild cognitive impairment (MCI), an early stage of AD, and ADRD significantly differs from healthy individuals [7, 8]. However, the significance of such changes in the prognosis of cognitive decline and ADRD remains elusive. In addition, the molecular mechanism of action by which the gut microbiota impacts brain health is not well understood. There is a strong connection between abnormal microbiota and increased chronic inflammation in ADRD affected older adults [6], however, the source of this inflammation remains less known. Our team recently formed a consortium called Microbiome in aging Gut and Brain (MiaGB) consortium study (Figure 1) focused on developing microbiome signature in older adults suffering age-related cognitive decline and ADRD, to understand the significance of microbiome signature in predicting the risk of cognitive decline and ADRD development in older adults. In addition, we also plan to develop a mechanistic understanding by which microbiota abnormalities contribute to age-related cognitive decline via influencing gut and systemic inflammation. Through this consortium we believe to establish proof-of-concept of microbiome utility as a biomarker for cognitive decline, which will pave the ways to design prevention and treatment strategies targeting microbiome in elderly.
